# Prevalence of polycystic ovary syndrome among adolescents depending on the adopted diagnostic criteria

**DOI:** 10.3389/fendo.2026.1785417

**Published:** 2026-04-16

**Authors:** Karolina Jakubowska-Kowal, Karolina Skrzyńska, Karolina Kowalczyk, Aneta Gawlik-Starzyk

**Affiliations:** 1 Department of Pediatrics and Pediatric Endocrinology, Medical University of Silesia, Katowice, Poland; 2 Department of Gynecology, Obstetrics and Gynecological Oncology, Medical University of Silesia, Katowice, Poland

**Keywords:** adolescent, hirsutism, hyperandrogenism, oligomenorrhea, polycystic ovary syndrome, prevalence

## Abstract

**Introduction:**

PCOS is a common endocrine disorder that impacts hormonal, metabolic, and reproductive health. Its prevalence is steadily increasing, including in the pediatric population, yet there is still a lack of research thoroughly examining the problem of teenage PCOS.

**Aim:**

The purpose of the study was to determine the prevalence and clinical-hormonal profile of PCOS among teenage patients with hyperandrogenism and/or menstrual disorders, depending on the adopted diagnostic criteria.

**Methods:**

An observational study was conducted on a group of 289 patients referred for diagnosis due to hyperandrogenism and/or menstrual disorders in the years 2019-2022. The study participant were assigned to appropriate groups depending on the diagnostic criteria met (patients with PCOS according to Ibanez, Peña, Rotterdam and patients without PCOS) and descriptive statistics and correlation studies were prepared.

**Results:**

The prevalence of polycystic ovary syndrome ranged from approximately 46% to 59%, depending on the diagnostic criteria applied. No statistically significant differences in clinical or hormonal parameters were observed between the three PCOS diagnostic groups (Ibáñez, Peña, and Rotterdam). However, patients meeting any PCOS definition differed significantly from those meeting no diagnostic criteria (NO PCOS group) in selected hormonal parameters: LH (p<0.001), total testosterone (p<0.001), androstenedione (p=0.003), and ovarian volume, consistent with established PCOS pathophysiology. The most frequent menstrual abnormality was “oligomenorrhea >45 days”. Clinical hyperandrogenism was more common than biochemical hyperandrogenism (88.1%–91.5% vs. 54.9%–55.3%), indicating that clinical signs of androgen excess predominate in adolescents. Acne occurred in around 70% of girls with polycystic ovary syndrome and in less than 40% of those without it. Across all diagnostic definitions, ovarian volume was consistently higher in patients diagnosed with the syndrome, even in cases not meeting the traditional criteria for polycystic ovarian morphology.

**Conclusion:**

The high prevalence of polycystic ovary syndrome among adolescents underscores the need for standardized diagnostic approaches. Improved understanding of clinical and hormonal profiles in this population can facilitate earlier and more accurate diagnosis, enabling timely preventive and therapeutic interventions to reduce long-term metabolic and reproductive complications.

## 1 Introduction

Polycystic Ovary Syndrome (PCOS) is a common condition affecting a significant number of women of reproductive age worldwide. Recent meta-analyses indicate that the global prevalence of PCOS may be as high as 9.2%. The reported prevalence varies from 5.5% to 11.5%, depending on the diagnostic criteria applied (1). Among adolescent females, the prevalence also depends on the criteria used and is estimated to range between 3.5% and 11% (2). In adult women, the most widely used diagnostic approach is based on the Rotterdam criteria, which require the presence of at least two out of three of the following features for diagnosis: 1) clinical and/or biochemical hyperandrogenism, 2) ovulatory dysfunction, 3) polycystic ovarian morphology on ultrasound or elevated anti-Müllerian hormone (AMH) levels (3). Given that some of the features included in these criteria may be physiological during adolescence, applying adult diagnostic criteria to adolescent populations may result in overdiagnosis. Therefore, specific diagnostic guidelines for adolescents have been proposed, including those by Ibáñez et al. (2017) and Peña et al. (2020) (4, 5). While these two sets of criteria differ slightly, both require the presence of menstrual irregularities and clinical or biochemical evidence of hyperandrogenism for diagnosis. PCOS in adolescent patients has become an increasingly frequent subject of clinical research; however, our understanding of this specific population remains limited. Considering the rising number of diagnoses and the potential long-term complications associated with PCOS, it is essential to continue deepening our knowledge in order to improve the quality and accuracy of early diagnosis (6, 7). The purpose of the study was to determine, using different diagnostic criteria, the prevalence and clinical-hormonal profile of PCOS in adolescents hospitalized due to hyperandrogenism and/or menstrual irregularities.

## 2 Materials and methods

We conducted an retrospective analysis of medical records of 289 patients referred to the Department of Pediatrics and Pediatric Endocrinology, due to hyperandrogenism and/or menstrual irregularities between June 2019 and December 2022. The study included all eligible patients identified in the medical records during the study period; therefore, no *a priori* sample size calculation was performed.

The Bioethics Committee of the Silesian Medical University in Katowice was consulted and determined that the retrospective analysis of existing medical records does not constitute a medical experiment and therefore does not require formal ethics committee review or approval under applicable regulations. (BNW/NWN/0052/KB/236/25).

A STROBE flow diagram of participant selection is presented in **Supplementary Figure 1**.

During the medical interview, patients answered questions about the minimum, maximum and typical length of their menstrual cycle and any other menstrual disorders they experienced. The definitions of the types of menstrual disorders were adopted in accordance with those presented in the criteria presented in **Table 1**.

**Table 1:** Summary of diagnostic criteria used in the study.

Diagnostic criterion	Ibanez L et al. 2017 (4)	Peña AS et al. 2020 (5)	Rotterdam 2023 (3)
Menstrual disturbances	Irregular menses/oligomenorrhea: oligomenorrhea (menstrual cycles longer than 45 days) and secondary amenorrhea (absence of cycles for more than 3 months) beyond 2 years after menarche; dysfunctional uterine bleeding - cycles shorter than 21 days or lasting more than 7 days; primary amenorrhea in girls with completed puberty	Irregular menstrual cycles: > 90 days for any one cycle (> 1 year post-menarche), cycles < 21 or > 45 days (> 1 to < 3 years post-menarche); cycles < 21 or > 35 days (> 3 years post-menarche) and primary amenorrhea by age 15 or > 3 years post-thelarche	Irregular menstrual cycles are defined as: 1 to <3 years post menarche: <21 or >45 days; 3 years post menarche to perimenopause: <21 or >35 days or <8 cycles per year; 1 year post menarche >90 days for any one cycle; Primary amenorrhea by age 15 or >3 years post thelarche (breast development)
Clinical or biochemical hyperandrogenism	Clinical hyperandrogenism - progressive hirsutism (modified Ferriman-Gellwey score >=8) and/or moderate to severe acne	Clinical hyperandrogenism - hirsutism defined as modified Ferriman-Gellwey score >=4–6 (depending on ethnicity; in our study of the Polish population the cut-off point was adopted as 4) and/or severe acne; Rarely alopecia	Clinical hyperandrogenism - hirsutism defined as modified Ferriman-Gellwey score >=4–6 (depending on ethnicity; in our study of the Polish population the cut-off point was adopted as 4) and/or severe acne
	Biochemical hyperandrogenism - elevations of total and/or free testosterone. No clear cut off for testosterone given. For the purposes of the study, the cut-off point for testosterone was 55 ng/dL.	Biochemical hyperandrogenism - calculated free testosterone, free androgen index or bioavailable testosterone. No cut offs given. For the purposes of the study, the cut-off point for testosterone was 55 ng/dL	Biochemical hyperandrogenism - calculated free testosterone, free androgen index or calculated bioavailable testosterone (Interpretation of androgen levels needs to be guided by the reference ranges of the laboratory used). For the purposes of the study, the cut-off point for testosterone was 55 ng/dL
Polycystic Ovarian Morphology (PCOM)	Pelvic ultrasound is not needed for PCOS diagnosis in adolescents	Pelvic ultrasound not recommended for diagnosis of PCOS within 8 years post menarche	PCOM - In transabdominal ultrasound - ovarian volume ≥10 ml on either ovary or follicle number per ovary (FNPO) ≥ 20 or follicle number per section (FNPS) ≥ 10 in at least one ovary. AMH (serum anti-mullerian hormone) can be used to diagnose PCOM as an alternative to ultrasound examination (no clear cut-off point for AMH values). PCOM diagnosis is not recommended for teenage patients.
PCOS diagnosis criteria	Diagnosis of PCOS if the presence of both (a) irregular menstrual cycles and ovulatory dysfunction as well as (b) hyperandrogenism clinical or biochemical.		Two of (a) oligo- or anovulation, (b) clinical and/or biochemical hyperandrogenism, (c) polycystic ovaries on ultrasound.

We used the modified Ferriman-Gallwey (mFG) scale to assess hirsutism (8). During the physical examination, 9 body regions were assessed (upper lip, chin, chest, upper back, lower back, upper abdomen, lower abdomen, upper arms, thighs), and each location was scored from 0 (no growth of terminal hair) to 4 (extensive hair growth). The total score was recorded. In cases where prior hair removal precluded objective assessment of hirsutism using the modified Ferriman–Gallwey (mFG) score on the day of examination, a detailed patient interview was conducted regarding the presence, distribution, and pattern of terminal hair growth. Based on this subjective assessment, patients were classified as “hirsutism present on subjective examination due to prior hair removal” if the reported hair growth met clinical criteria for hirsutism. This approach allowed us to account for signs of hirsutism even when mFG scoring was not possible at the time of evaluation. The interview also included questions about the presence and severity of acne and alopecia. Acne was defined as the presence of inflammatory and/or comedonal lesions of the face, chest, or back, as documented by the examining physician in the medical record. Severity grading using a validated scale (e.g., Global Acne Grading System, Investigator Global Assessment) was not routinely performed at the time of data collection; accordingly, acne is reported as a binary variable (present/absent) and no quantitative severity comparisons are made. This constitutes a recognized limitation of retrospective studies in this field.

Total testosterone was measured in a single certified laboratory using routine clinical assays. Given the lack of validated adolescent-specific testosterone cutoffs, we applied a pragmatic total testosterone threshold of 55 ng/dL, reflecting the upper range of values commonly used in previous studies of adolescent hyperandrogenism.

Pelvic ultrasound examinations were performed transabdominally as part of routine clinical care using a Siemens Medical Solutions USA, Inc. system equipped with a convex transducer (frequency 3.5–5.0 MHz). All examinations were performed or directly supervised by a single experienced physician, ensuring operator consistency throughout the study period. Ovarian volume was calculated using the simplified ellipsoid formula: V = 0.523 × D1 × D2 × D3, where D1, D2, and D3 represent the three perpendicular diameters measured in millimetres. Cycle phase at the time of ultrasound was not standardised, as examinations were performed during routine inpatient admission rather than at a pre-specified cycle time point; this represents a limitation of the study and all ovarian morphology findings should be interpreted as exploratory.

Three different diagnostic criteria were applied– the Rotterdam criteria, primarily intended for the adult population, and two sets of criteria specifically developed for adolescents: those proposed by Ibáñez et al. and by Peña et al. (3–5) Details of the diagnostic criteria used in the study are presented in **Table 1**.

Each patient was assigned to one or more of the following groups:

-IBANEZ, if met the criteria proposed by Ibáñez et al. (5);
-PENA, if fulfilled the criteria proposed by Peña et al. (4);
-ROTTERDAM, if met the Rotterdam criteria (3);
-NO PCOS, if did not meet any of the above-mentioned diagnostic criteria for PCOS,
-IBANEZ/PENA/ROTTERDAM, if met any PCOS diagnosis criteria

Each patient could be classified into more than one group, for instance, if she simultaneously met the diagnostic criteria of two different authors. The Peña and Rotterdam guidelines differ primarily in whether or not they include the PCOM criterion. Although the Rotterdam criteria, including the PCOM criterion, are not recommended as primary diagnostic tools in adolescents by current international guidelines, they continue to be used in clinical practice and are included in this study for comparative and exploratory purposes only, to quantify the diagnostic discordance between adult-derived and adolescent-specific criteria. Results for the Rotterdam group should therefore be interpreted as exploratory and hypothesis-generating rather than confirmatory. All primary clinical conclusions in this study are based on the Ibáñez and Peña criteria.

Proportions are reported with 95% confidence intervals calculated using the Wilson score method.

Exclusion criteria comprised eating disorders (anorexia nervosa, bulimia), hyperprolactinemia (prolactin [PRL] ≥721 mIU/l), adrenal disorders (17-hydroxyprogesterone [17OHP] ≥30 nmol/l, in patients with 17OHP between 6-29.9 nmol/l, urine steroid profile results suggestive of congenital adrenal hyperplasia), and the use of medications known to influence sex steroids in the last 3 months (oral contraceptives, glucocorticosteroids, antiandrogens, aromatase inhibitors, metformin, anticonvulsants, etc.).

In all patients, the following data were analyzed:

- clinical profile: age on admission [years], age of menarche [years], gynecological age [years], minimum, maximum and typical length of menstrual cycle [days], hirsutism assessed according to the modified Ferriman-Gallwey scale [points] (8);
- hormonal profile: total testosterone - T [ng/dl], luteinizing hormone - LH [IU/l], follicle stimulating hormone - FSH [IU/l], dehydroepiandrosterone sulfate - DHEAS [µmol/l], androstenedione – A [nmol/l], 17-hydroxyprogesterone – 17-OHP [nmol/l]; The provided hormone reference ranges are consistent with the standards of the laboratory performing the tests and are appropriate for the patient’s age and phase of the menstrual cycle.
- pelvic ultrasound – dimensions [mm] and volume [ml] of ovaries.

### 2.1 Statistical analysis

Statistical analyses were performed using Statistica software, version 9.1. All statistical analyses were performed using the available data from each variable. Due to missing data for some variables, each analysis was conducted on a different number of patients; the sample size (N) is reported alongside each correlation coefficient. In cases where outliers were identified for certain variables, analyses were conducted excluding these outlier observations.

Descriptive statistics were calculated to summarize the characteristics of the study population. Continuous variables were reported as mean ± standard deviation (SD) or median with interquartile range (IQR), depending on their distribution, while categorical variables were summarized as counts and percentages. These analyses provided an overview of patient demographics, clinical parameters, and other relevant study variables.

Before applying statistical tests, the assumptions for parametric analyses (normality and homogeneity of variance) were evaluated where possible. For variables that did not meet these assumptions, non-parametric tests were used.

Correlations between quantitative variables were assessed using correlation coefficients appropriate to data distribution. Correlation results are reported as the correlation coefficient (r) with the associated p-value (p), with r serving as a measure of effect size.

Formal normality testing (Shapiro-Wilk) revealed significant non-normality in 12 of 13 continuous variables; therefore, all quantitative correlations were assessed using Spearman’s rank correlation coefficient (r^s^). Ninety-five percent confidence intervals for r^s^ were estimated by 1, 000 bootstrap iterations (percentile method). To control the familywise error rate, the Holm-Bonferroni sequential correction was applied within each family of tests: 66 pairs in the correlation matrix (**Table 6**) and 12 qualitative-quantitative comparisons (**Table 7**). Corrected p-values (p_Holm) are reported alongside raw p-values.

Differences between groups in continuous variables were assessed using the Mann–Whitney U test. For smaller sample sizes, the U statistic is reported, whereas for larger samples, the standardized Z value is presented. Associations between categorical variables were examined using the Chi-square test, with Yates’ continuity correction applied when appropriate.

Statistical significance was defined as p_Holm < 0.05.

## 3 Results

### 3.1 Descriptive characteristics

289 individuals met the inclusion criteria. Clinical and hormonal profile obtained during hospitalization for the entire cohort are presented in **Table 2**.

**Table 2:** Clinical and hormonal profile of patients with hyperandrogenism and/or menstrual disorders.

Variables	N	M (95%CI)	SD	Min	Max	Q1	Me	Q3
Age on admission [years]	289	16.2 (16.04–16.36)	1.4	10.8	17.9	15.5	16.4	17.2
Menarche age [years]	265	12.1 (11.92–12.28)	1.5	8.0	16.0	11.0	12.0	13.0
Gynecological age [years]	265	4.2 (4.01–4.39)	1.6	0.5	9.1	3.0	4.1	5.2
Hirsutism [Ferriman – Gallwey score]	102	9.3 (8.25–10.35)	5.4	0.0	24.0	6.0	8.0	12.0
Hirsutism (present on subjective examination) [yes-1]	N=47 (16.3%)							
Acne (present on subjective examination) [yes-1]	N=163 (56.4%)							
LH [IU/l] (norms: 1-39.3)	288	10.5 (9.51–11.49)	8.6	0.3	54.1	4, 4	8.0	13.0
FSH [IU/l] (norms: 1.6-9.8)	288	6.1 (5.18–7.02)	8.0	0.7	113.0	3.6	5.5	7.0
Total testosterone [ng/dl] (norms 5-40)	283	53.6 (50.69–56.51)	25.0	2.5	242.8	36.8	52.3	66.9
DHEA-S [µmol/l] (norms 3.0-9.9)	282	8.6 (8.13–9.07)	4.0	0.4	21.6	5.3	7.9	11.2
Androstenedione [nmol/l]	187	15.4 (14.55–16.25)	5.9	3.5	33.5	11.2	15.0	19.2
17-hydroxyprogesterone [nmol/l]	188	4.5 (3.99–5.01)	3.6	0.3	19.1	1.8	3.3	6.7
Right ovary, dimension no. 1 [mm]	245	32.9 (31.92–33.88)	7.8	0.0	52.0	28.0	33.0	38.0
Right ovary, dimension no. 2 [mm]	245	19.0 (18.31–19.69)	5.5	0.0	40.0	16.0	19.0	22.0
Right ovary volume [ml]	244	6.0 (5.56–6.44)	3.5	0.0	27.6	3.5	5.3	7.9
Left ovary, dimension no. 1 [mm]	242	31.3 (30.33–32.27)	7.7	4.3	57.0	27.0	30.0	35.0
Left ovary, dimension no. 2 [mm]	242	19.0 (18.40–19.60)	4.8	1.9	40.0	16.0	19.0	22.0
Left ovary volume [ml]	238	5.8 (5.32–6.28)	3.8	0.2	27.0	3.2	5.1	7.5

[N, number of data values; M (95% CI), mean value with 95% confidence interval; Me, median; Min, minimum value; Max, maximum value; Q1, lower quartile; Q3, upper quartile, SD, standard deviation; Data are presented as mean (95% confidence interval) and standard deviation (SD). Median (Me) and interquartile range (Q1–Q3) are shown for descriptive purposes].

The proportion of adolescents meeting diagnostic criteria for PCOS in this referred cohort was 46.4% (95% CI 40.7–52.1) according to the Ibáñez criteria (IBANEZ group), 52.9% (95% CI 47.2–58.6) according to the Peña criteria (PENA group), and 58.8% (95% CI 53.1–64.3) according to the Rotterdam criteria (ROTTERDAM group).

**Figure 1:**
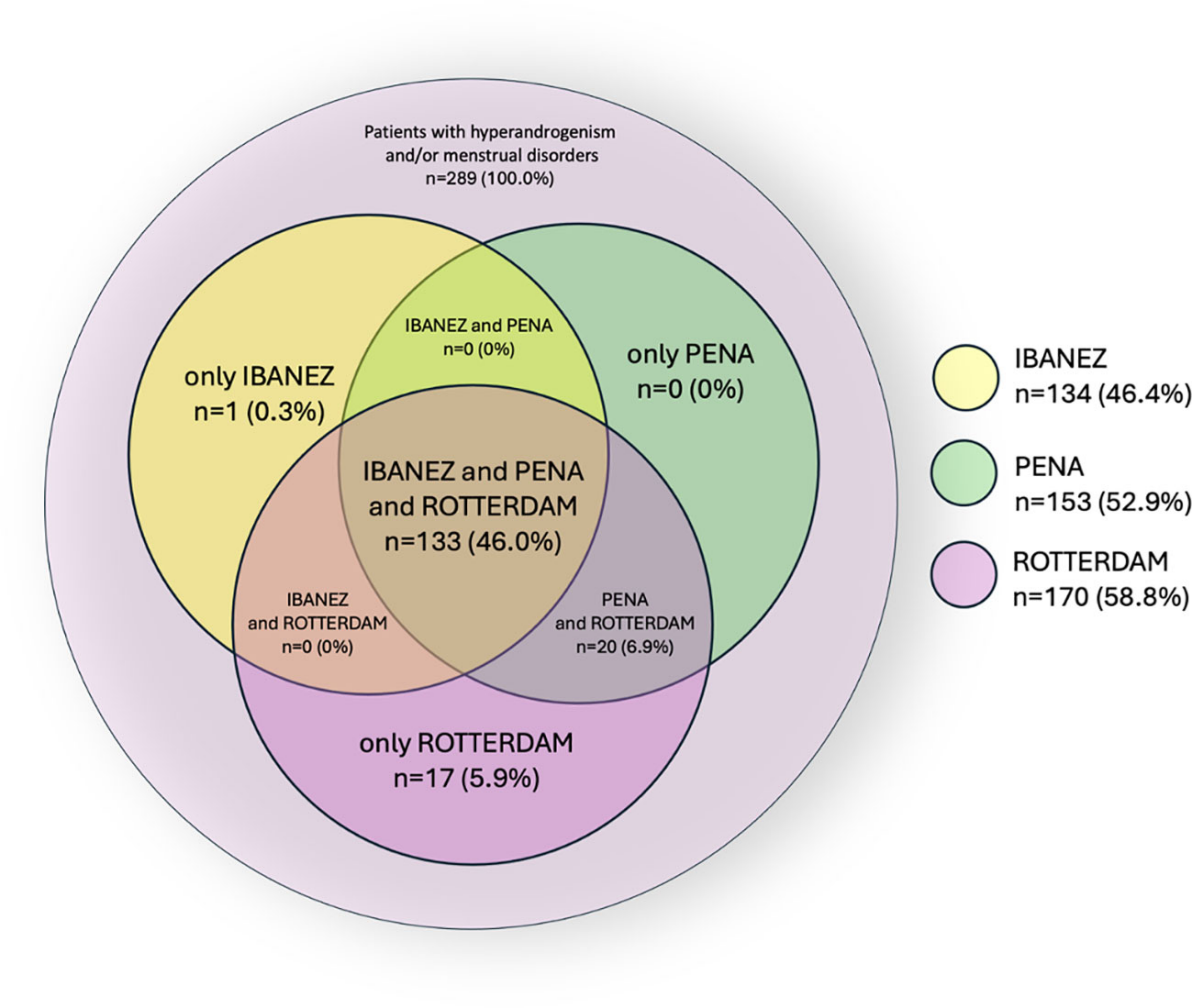
Prevalence PCOS among patients depending on the adopted diagnosis criteria.

In 40.8% of patients, none of the applied diagnostic criteria for PCOS were met (NO PCOS group). The prevalence of PCOS in the studied population, according to the different diagnostic criteria, is illustrated in **Figure 1**.

**Table 3** presents the frequency of clinical features relevant to PCOS diagnosis, as required by the applied diagnostic criteria, across the different groups.

**Table 3:** Prevalence of characteristics from the subjective history in individual groups [%] .

Characteristics		Study group	NO PCOS	IBANEZ	PENA	ROTTERDAM
*menarche*	Minimum 1 year after menarche	90.7%	88.1%	91.0%	91.5%	92.4%
	Minimum 2 years after menarche	85.5%	83.9%	88.8%	85.6%	86.5%
*menstrual cycle*	Regular cycles	20.1%	39.0%	0.8%	1.3%	7.1%
	Oligomenorrhea >35days	6.6%	6.8%	0.0	7.2%	6.5%
	Oligomenorrhea >45days	50.9%	20.3%	85.1%	79.1%	71.8%
	Polymenorrhea	12.1%	5.9%	18.7%	17.0%	16.5%
	Primary amenorrhea	8.3%	9.3%	9.0%	8.5%	7.7%
	Secondary amenorrhea	46.4%	37.3%	59.0%	56.9%	52.9%
	Menorrhagia	2.8%	1.7%	3.7%	3.2%	3.5%
	Menometrorrhagia	6.6%	5.9%	7.5%	7.2%	7.1%
	Dysmenorrhea	4.5%	4.2%	3.7%	4.6%	4.7%
*clinical hyperandrogenism*	Acne	56.4%	38.1%	70.2%	69.9%	68.8%
	Hirsutism present on subjective examination, not assessable due to depilation	16.3%	9.3%	20.2%	20.3%	21.2%

**Table 4** summarizes the frequency of individual diagnostic criteria met by patients in each group.

**Table 4:** Frequency of individual diagnostic criteria in the patient groups "IBANEZ", "PENA", "ROTTERDAM".

Diagnostic criterion	IBANEZ	PENA	ROTTERDAM
Irregular menstrual cycles	100.0%	100.0%	91.8%
Clinical Hyperandrogenism	88.1%	91.5%	89.4%
Biochemical Hyperandrogenism	55.2%	54.9%	55.3%
PCOM	–	–	24.1%

PCOM, Polycystic Ovarian Morphology.

Sensitivity analyses excluding patients with gynecological age < 1 year (n=3) and < 2 years (n=18) from menarche did not materially alter the observed PCOS prevalence (maximum change: 2.2 percentage points) or hormonal distributions. These results are presented in **Supplementary Table S2**.

Of the 289 patients, 47 (16.3%) were classified as having hirsutism present on subjective examination due to prior hair removal (i.e., objective mFG scoring was not feasible). A sensitivity analysis excluding these 47 patients showed a reduction in PCOS prevalence of 2.2–3.4 percentage points depending on the diagnostic criterion applied, and a reduction in clinical hyperandrogenism of approximately 9–10 percentage points, without altering the qualitative conclusions of the study (**Supplementary Table S3**).

### 3.2 Comparative study

**Figure 2:**
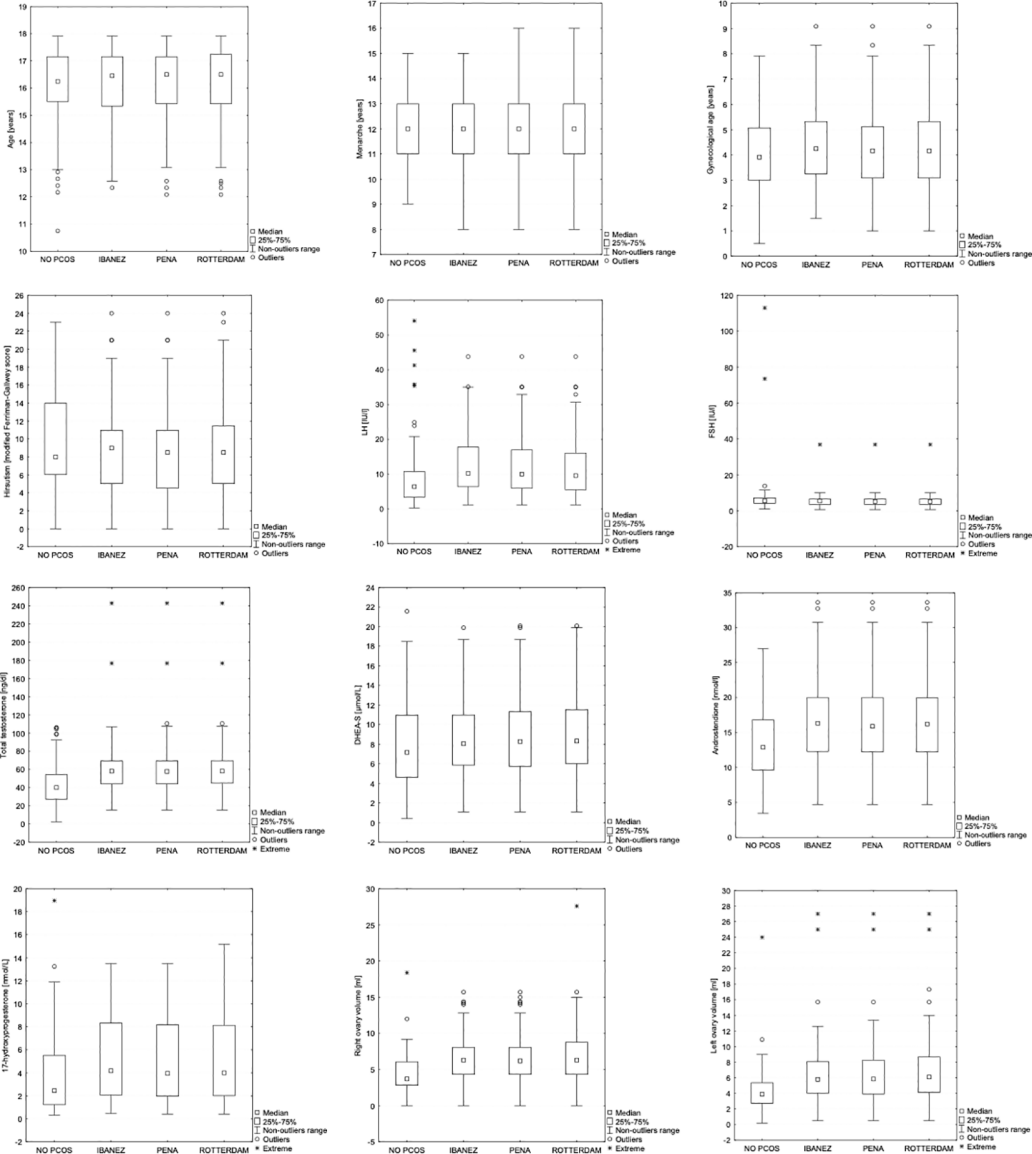
The comparison of clinical and hormonal profiles between “NO PCOS”, “IBANEZ”, “PENA” and “ROTTERDAM” groups - graphic presentation.

No significant differences in clinical or hormonal characteristics were detected among the three PCOS diagnostic groups defined according to the Ibáñez, Peña, and Rotterdam criteria. In contrast, adolescents fulfilling any of the PCOS diagnostic frameworks differed significantly from those not meeting diagnostic criteria (NO PCOS group) in several hormonal parameters, including LH (p<0.001), total testosterone (p<0.001), androstenedione (p=0.003), and ovarian volume, findings that align with the known pathophysiological features of PCOS. Additionally, serum 17-hydroxyprogesterone concentrations showed statistically significant differences between the NO PCOS group and the Ibáñez and Rotterdam groups, whereas no significant difference was observed for the Peña group. The comparison of clinical and hormonal profiles between groups are presented in **Table 5** and **Figure 2**.

**Table 5:** The comparison of clinical and hormonal profiles between “NO PCOS”, “IBANEZ”, “PENA” and “ROTTERDAM” groups.

Variable	Study groups	N	M (95% CI)	SD	Q1	Me	Q3	Group comparison
Age [years]	I) NO PCOS	118	16.13 (15.88–16.38)	1.39	15.50	16.25	17.17	H = 0.50 p = 0.92
	II) IBANEZ	134	16.16 (15.94–16.38)	1.32	15.33	16.46	17.17	
	III) PENA	153	16.16 (15.95–16.37)	1.33	15.42	16.50	17.17	
	IV) ROTTERDAM	170	16.21 (16.01–16.41)	1.35	15.42	16.50	17.25	
Menarche [years]	I) NO PCOS	107	12.05 (11.80–12.30)	1.34	11.00	12.00	13.00	H = 0.12 p = 0.99
	II) IBANEZ	122	11.96 (11.71–12.21)	1.44	11.00	12.00	13.00	
	III) PENA	140	12.04 (11.78–12.30)	1.58	11.00	12.00	13.00	
	IV) ROTTERDAM	157	12.06 (11.81–12.31)	1.57	11.00	12.00	13.00	
Gynecological age [years]	I) NO PCOS	107	4.02 (3.72–4.32)	1.58	3.00	3.92	5.08	H = 1.49 p = 0.69
	II) IBANEZ	122	4.31 (4.04–4.58)	1.50	3.25	4.25	5.33	
	III) PENA	140	4.21 (3.95–4.47)	1.59	3.08	4.17	5.13	
	IV) ROTTERDAM	157	4.24 (3.98–4.50)	1.63	3.08	4.17	5.33	
Hirsutism [modified Ferriman--Gallwey score]	I) NO PCOS	29	9.38 (7.25–11.51)	5.84	6.00	8.00	14.00	H = 0.10 p = 0.99
	II) IBANEZ	63	9.16 (7.89–10.43)	5.14	5.00	9.00	11.00	
	III) PENA	68	9.06 (7.84–10.28)	5.15	4.50	8.50	11.00	
	IV) ROTTERDAM	72	9.24 (8.01–10.47)	5.30	5.00	8.50	11.50	
LH [IU/l]	I) NO PCOS	118	8.83 (7.21–10.45)	8.97	3.24	6.37	10.80	**H = 26.44** **p < 0.001** **I < (II, III, IV)**
	II) IBANEZ	133	12.56 (11.08–14.04)	8.71	6.29	10.20	17.80	
	III) PENA	152	12.23 (10.89–13.57)	8.44	5.83	9.95	17.00	
	IV) ROTTERDAM	169	11.70 (10.45–12.95)	8.25	5.42	9.53	16.10	
FSH [IU/l]	I) NO PCOS	118	7.17 (5.02–9.32)	11.88	4.04	5.54	7.41	H = 3.62 p = 0.31
	II) IBANEZ	133	5.39 (4.81–5.97)	3.40	3.48	5.47	6.67	
	III) PENA	152	5.40 (4.88–5.92)	3.27	3.48	5.19	6.74	
	IV) ROTTERDAM	169	5.37 (4.89–5.85)	3.17	3.48	5.17	6.73	
Total testosterone [ng/dl]	I) NO PCOS	115	44.30 (40.19–48.41)	22.47	27.21	40.13	54.72	**H = 48.15** **p < 0.001** **I < (II, III, IV)**
	II) IBANEZ	132	59.78 (55.36–64.20)	25.85	43.82	58.16	69.71	
	III) PENA	151	59.84 (55.76–63.92)	25.59	43.86	57.80	69.74	
	IV) ROTTERDAM	167	60.08 (56.32–63.84)	24.68	44.66	58.25	70.04	
DHEA-S [µmol/L]	I) NO PCOS	114	7.93 (7.18–8.68)	4.12	4.61	7.18	10.99	H = 6.86 p = 0.08
	II) IBANEZ	132	8.64 (8.01–9.27)	3.66	5.85	8.06	11.02	
	III) PENA	151	8.73 (8.13–9.33)	3.79	5.73	8.28	11.34	
	IV) ROTTERDAM	167	8.96 (8.38–9.54)	3.82	6.00	8.33	11.56	
Androstendione [nmol/L]	I) NO PCOS	72	13.55 (12.32–14.78)	5.31	9.60	12.92	4.83	**H = 13.81** **p = 0.003** **I < (II, III, IV)**
	II) IBANEZ	94	16.52 (15.33–17.71)	5.87	12.26	16.34	5.74	
	III) PENA	103	16.41 (15.27–17.55)	5.90	12.19	15.89	5.74	
	IV) ROTTERDAM	114	16.41 (15.33–17.49)	5.87	12.19	16.24	5.73	
17-hydroxy-progesterone [nmol/L]	I) NO PCOS	72	3.72 (2.94–4.50)	3.39	1.21	2.45	5.54	**H = 10.30** **p = 0.016** **I < II, I < IV**
	II) IBANEZ	95	5.08 (4.37–5.79)	3.54	2.06	4.18	8.32	
	III) PENA	104	4.90 (4.22–5.58)	3.54	1.97	3.96	8.17	
	IV) ROTTERDAM	115	4.99 (4.33–5.65)	3.60	2.00	3.99	8.14	
Right ovary volume [ml]	I) NO PCOS	95	4.57 (4.01–5.13)	2.80	2.80	3.70	6.10	**H = 35.37** **p <0.001** **I < (II, III, IV)**
	II) IBANEZ	116	6.50 (5.93–7.07)	3.12	4.30	6.30	8.10	
	III) PENA	131	6.45 (5.91–6.99)	3.14	4.30	6.20	8.10	
	IV) ROTTERDAM	148	6.86 (6.26–7.46)	3.70	4.30	6, 30	8.80	
Left ovary volume [ml]	I) NO PCOS	96	4.39 (3.82–4.96)	2.84	2.70	3.90	5.40	**H = 40.42** **p <0.001** **I < (II, III, IV)**
	II) IBANEZ	109	6.44 (5.70–7.18)	3.93	4.00	5.80	8.10	
	III) PENA	124	6.44 (5.76–7.12)	3.87	3.90	5.85	8.30	
	IV) ROTTERDAM	141	6.86 (6.20–7.52)	4.00	4.10	6.10	8.70	

(N, number of data values; M (95% CI), mean value with 95% confidence interval; SD, standard deviation; Q1, lower quartile; Me, median; Q3, upper quartile; H, Kruskal-Wallis test; p, probability value).

Statistically significant information is bolded.

### 3.3 Correlation of quantitative variables

The results of the correlation analysis of quantitative variables in the IBANEZ/PENA/ROTTERDAM group are presented in **Table 6**. All pairwise correlations were tested; however, for clarity, only statistically significant correlations are presented in the table.

**Table 6:** Correlations of quantitative variables in the IBANEZ/PENA/ROTTERDAM group.

Variable 1	Variable 2	r^s^	n	p	95% CI	p_Holm
Menarche age [years]	Gynecological age [years]	-0.662	158	<0.001	[-0.741, -0.564]	<0.001
Age on admission [years]	Gynecological age [years]	0.450	158	<0.001	[0.317, 0.567]	<0.001
DHEA-S [µmol/l]	Andro-stenedione [nmol/m]	0.432	115	<0.001	[0.270, 0.570]	<0.001
Age on admission [years]	Menarche age [years]	0.371	158	<0.001	[0.229, 0.499]	<0.001
LH [IU/l]	Total testosterone [ng/dl]	0.345	168	<0.001	[0.204, 0.471]	<0.001
Total testosterone [ng/dl]	Andro-stenedione [nmol/m]	0.409	115	<0.001	[0.244, 0.551]	<0.001
Androstenedione [nmol/l]	17-hydroxyproges- terone [nmol/l]	0.390	115	<0.001	[0.223, 0.535]	0.001
Total testosterone [ng/dl]	DHEA-S [µmol/l]	0.310	168	<0.001	[0.167, 0.441]	0.003
LH [IU/l]	FSH [IU/l]	0.290	170	<0.001	[0.146, 0.422]	0.007
Right ovary volume [ml]	Left ovary volume [ml]	0.284	142	<0.001	[0.125, 0.429]	0.035

(r^s^, Spearman rank correlation coefficient; n, number of observations; p, raw p-value; 95% CI, 95% confidence interval (1, 000 bootstrap iterations); p_Holm, Holm-Bonferroni corrected p-value, statistical significance was defined as p_Holm<0.05).

### 3.4 Correlation of qualitative and quantitative variables

We examined the correlations between all quantitative and qualitative variables, but for clarity, only those with statistically significant results were presented in the paper.

Lower LH concentrations were associated with regular menstrual cycles, whereas elevated LH levels were observed in patients with secondary amenorrhea. Lower testosterone concentrations were associated with the presence of polymenorrhea and, somewhat unexpectedly, with the occurrence of acne. (**Table 7**).

**Table 7:** Associations between clinical (qualitative) features and quantitative variables in the IBANEZ/PENA/ROTTERDAM group (Mann-Whitney U / Z-test).

Analyzed variable	Regular cycles	M (95% CI)	SD	Min	Q1	Me	Q3	Max	Group comparison
LH [IU/l]	no (n=158)	12.1 (10.81–13.39)	8.3	1.1	5.7	9.6	16.9	43.8	U = 467.0 p = 0.003 p_Holm = 0.027
	yes (n=12)	5.8 (4.10–7.50)	3.0	1.6	3.7	5.5	7.1	12.0	
DHEA-S [µmol/l]	no (n=156)	8.7 (8.12–9.28)	3.7	1.1	5.8	8.2	11.3	20.1	U = 460.0 p = 0.003 p_Holm = 0.027
	yes (n=12)	12.1 (10.07–14.13)	3.6	6.4	9.0	12.9	14.8	17.3	
Analyzed variable	Polymeno-rrhea	M (95% CI)	SD	Min	Q1	Me	Q3	Max	Group comparison
Testosterone [ng/dl]	no (n=141)	62.1 (57.87–66.33)	25.6	15.6	47.2	59.3	71.9	242.8	Z = 3.058 p = 0.002 p_Holm = 0.020
	yes (n=27)	48.3 (42.56–54.04)	15.2	21.5	38.6	48.3	61.1	78.4	
Analyzed variable	Secondary amenorrhea	M (95% CI)	SD	Min	Q1	Me	Q3	Max	Group comparison
LH [IU/l]	no (n=80)	9.2 (7.91–10.49)	5.9	1.1	4.9	7.9	11.9	27.5	Z = -3.362 p = 0.001 p_Holm = 0.011
	yes (n=89)	14.0 (12.05–15.95)	9.4	2.1	7.3	11.5	19.7	43.8	
Analyzed variable	Acne	M (95% CI)	SD	Min	Q1	Me	Q3	Max	Group comparison
Testosterone [ng/dl]	no (n=52)	63.9 (59.44–68.36)	16.4	15.6	56.9	66.5	73.9	95.3	Z = 3.97 p < 0.001 p_Holm = 0.001
	yes (n=116)	58.2 (53.21–63.19)	27.4	20.8	41.3	53.6	66.6	242.8	

(n, number of data values; M (95% CI). mean value with 95% confidence interval; SD, standard deviation; Min, minimum value; Q1, lower quartile; Me. median; Q3, upper quartile; Max, maximum value; p, raw p-value; U, Mann-Whitney U test; Z. Z-test; p_Holm, Holm-Bonferroni corrected p-value, statistical significance was defined as p_Holm<0.05).

## 4 Discussion

### 4.1 PCOS prevalence and criteria comparison

This study is the first of its kind in a population of pediatric PCOS patients. The prevalence of PCOS among adolescents referred for hospital diagnosis ranged from approximately 46% to 59% (depending on the diagnostic criteria applied). It is important to emphasize that these figures do not reflect the prevalence of PCOS in the general population, but rather in a selected population of adolescent patients presenting with hyperandrogenism and/or menstrual irregularities.

Patients from the study group were referred for in-hospital diagnostic evaluation by pediatric endocrinology specialists, who—owing to their clinical experience—were able to objectively assess the severity of reported symptoms and the clinical profile of each patient, enabling them to accurately identify individuals requiring further diagnostic work-up. Further evaluation at the Department of Pediatric Endocrinology, including a comprehensive medical history, clinical examination, and laboratory testing, confirmed the appropriateness of referral in a significant proportion of cases. Our study shows that in as many as half of patients suspected of having PCOS, this syndrome was confirmed.

The increasing incidence of PCOS in adolescents reported in studies as well as the results of our study highlight the importance of tailored diagnostic approaches and increased awareness among clinicians treating this age group (2, 9–17).

Comparison of the three PCOS diagnostic groups (Ibáñez, Peña, and Rotterdam) showed no statistically significant differences in any clinical or hormonal variable between these groups (**Table 5**; **Figure 2**), confirming the clinical equivalence of the three frameworks in this referred cohort. Importantly, this finding refers to comparisons among the three PCOS-positive groups and should not be confused with the comparison between PCOS-positive patients (under any criterion) and the NO PCOS group, which revealed significant differences in LH (p<0.001), total testosterone (p<0.001), androstenedione (p=0.003), and ovarian volume, as discussed in section 4.4. The Rotterdam analysis is presented for comparative and exploratory purposes; the Rotterdam group criteria, including PCOM, are not recommended as standalone diagnostic tools in adolescents per current guidelines.

### 4.2 Hyperandrogenism

In our study, the prevalence of clinical hirsutism was observed in 88.1% to 91.5% of patients with PCOS, which corresponds to the trend from data available in studies of the adult population (18). Furthermore, clinical hirsutism was diagnosed significantly more frequently in the PCOS group than biochemical hyperandrogenism (54.9%–55.3%) These findings highlight the importance of detailed history-taking regarding excessive hair growth and a thorough physical examination.

Interestingly, statistical analysis revealed similar Ferriman–Gallwey scores between patients with and without PCOS. This may be explained by the fact that hair distribution could not be properly assessed in many cases due to prior depilation, which limited the accuracy of clinical scoring. The problem of excessive hair growth and elevated androgen levels reported by patients were not always reflected in the Ferriman-Gallwey score. As a result, hirsutism assessment often relied on patient history and subjective reporting. In our study, such cases were labeled as “Hirsutism present on subjective examination, not assessable due to depilation.” This feature was observed approximately twice as frequently in girls with PCOS compared to those without the diagnosis. A 2012 study also noted the limited value of this indicator among teenage girls, but this study involved a small sample (19). Further research on this topic is necessary.

In our study, no correlations were found between the presence of hirsutism on physical examination and the analyzed variables. (**Table 7**) Similarly, in the correlation analysis of quantitative variables, no statistically significant associations with mFG scores were observed. (**Table 6**) These findings highlight the need for further investigation of biochemical and clinical markers of hirsutism in adolescent girls to enable more accurate and effective diagnosis.

Our correlation study showed that acne patients had lower testosterone levels. Further studies on a larger group of patients are needed to verify this surprising study result. (**Table 7**) Our work, like other available studies, shows a high prevalence of acne (68.8-70.2%) among teenage patients with PCOS (20). This suggests that we should pay particular attention to the group of adolescents presenting with this symptom, which is included in the diagnostic criteria as clinical hirsutism, and inquire more deeply about the possible presence of menstrual disorders.

### 4.3 Menstrual disorders

Although the association was statistically significant based on the raw p-value, it did not remain significant after Holm–Bonferroni correction for multiple comparisons for the correlations between the presence of a regular cycle and gynecological age as well as age at admission. Occurrence of “regular cycle” among PCOS patients statistically significantly correlated with lower LH and higher DHEA-S. It is important to note that, the guidelines for diagnosing PCOS in adolescents specify the required time from menarche to diagnosis. In the case of the Ibanez L et al., 2017 guidelines, this is beyond 2 years after menarche, while both Peña AS et al., 2020 and Rotterdam 2023 allow for diagnosis to begin as early as 1 year after menarche, depending on the type of cycle abnormalities present (3–5).

### 4.4 PCOM

It is worth noting that a comparison of patients with PCOS (in all IBANEZ, PENA, and ROTTERDAM groups) to those without the syndrome (NO PCOS) revealed statistically significant differences in LH, testosterone, androstenedione, and 17-hydroxyprogesterone levels, and interestingly, in ovarian volume. Regardless of the adopted diagnosis criteria, ovarian volume was larger in patients diagnosed with PCOS, even though they often did not meet the commonly accepted criteria for PCOM.

In our study, 17 patients were diagnosed with PCOS using the PCOM criteria from the Rotterdam guidelines (they did not meet the Peña or Ibanez guidelines). In addition to PCOM, these patients also exhibited clinical hyperandrogenism (4 cases), biochemical hyperandrogenism (2 cases), and both biochemical and clinical hyperandrogenism (8 cases). Further studies and follow-up are needed to determine whether these patients will develop PCOS in the future, or whether their polycystic ovarian ultrasound findings were merely a variant of the normal pubertal stage (21, 22).

It is necessary to carefully revisit ultrasound, which is safe, readily available and non-invasive, as an additional test in the diagnosis of adolescents, to be able to identify as many PCOS patients as possible and implement early lifestyle interventions. It should be remembered that the guidelines show that applying criteria for adults may lead to overdiagnosis, but we must be careful about the group of patients who, thanks to additional criteria, could be included in specialist care and observation for the possibility of developing PCOS in the future. Further research is needed to improve diagnostic accuracy and work toward refining and unifying adolescent-specific criteria to better identify true PCOS cases in this age group. It should be noted that as ultrasound in this study was not performed at a standardised menstrual cycle time point and cycle phase was not systematically recorded, all findings relating to ovarian volume and polycystic ovarian morphology must be regarded as exploratory and require confirmation in prospective studies using standardised cycle-timed ultrasound protocols.

### 4.5 Clinical implications

The diagnosis of PCOS was confirmed in almost half of the patients referred for further evaluation. This highlights the crucial role of physicians with various specialties working in the outpatient setting, who are the first to see patients and can identify patients requiring further evaluation for this syndrome.

The use of 95% confidence intervals allowed assessment of the clinical relevance of observed differences. While several variables showed statistically significant differences, many of these were associated with small absolute effect sizes and largely overlapping confidence intervals, suggesting limited clinical significance (e.g. age, menarche, FSH). In contrast, LH concentrations, total testosterone levels, and ovarian volumes demonstrated consistent shifts in confidence intervals across PCOS phenotypes compared with controls. These findings are directionally consistent with established PCOS pathophysiology; however, given the absence of BMI, insulin resistance indices, and lipid profile data in this retrospective analysis, we cannot exclude the possibility that obesity or metabolic dysregulation confounds some of these associations. These findings should be interpreted as hypothesis-generating, pending prospective studies with full metabolic profiling. For some hormonal parameters (e.g. DHEA-S, androstenedione), confidence intervals partially overlapped between groups, suggesting moderate group-level differences but limited discriminatory value at the individual patient level.

Laboratory test results do not always fully reveal the problem of hyperandrogenism in a previous patient, so acne and clinical hyperandrogenism are features that should be given special attention during the physical examination and medical history during a visit.

The use of ultrasound in the diagnosis of adolescents should be an area of ​​further research, as this non-invasive method could be a supportive tool to guide our diagnostic work in the appropriate direction.

The lack of significant differences between the profiles of patients who met the criteria of the individual guidelines emphasizes the need to establish a single, common diagnostic path, which will make the process easier and more transparent.

### 4.6 Limitations of the study

The retrospective, observational, cross-sectional design of the study, without a general population control group, precludes causal inference and limits the strength of comparative conclusions.

The study included girls presenting with menstrual disturbances and/or hyperandrogenism and therefore does not represent the general healthy adolescent female population. As a single-center, hospital-based study, this design may have introduced referral bias and precludes extrapolation of the reported proportions to the general population. However, adolescents who did not meet the diagnostic criteria for polycystic ovary syndrome served as the control group, enabling a clinically relevant comparison that reflects real-world diagnostic pathways.

Another limitation is the partial overlap between the IBANEZ, PENA, and ROTTERDAM diagnostic frameworks, as some patients met more than one set of criteria, which may introduce heterogeneity in group classification and should be considered when interpreting the results.

Not all data were complete, as some clinical information from patient interviews was unavailable and isolated laboratory measurements were missing in a subset of participants. In addition, objective assessment of hirsutism was frequently not feasible due to prior hair removal practices, which may have affected the evaluation of clinical hyperandrogenism.

This study did not systematically collect body mass index (BMI) or BMI standard deviation scores, insulin resistance indices (e.g., HOMA-IR, fasting insulin), or lipid profile data, as these variables were not uniformly available across the retrospective records. These are recognised confounders of androgen metabolism and ovarian morphology in adolescent PCOS. Their absence limits the ability to disentangle the independent contributions of obesity, insulin resistance, and intrinsic ovarian dysregulation to the observed hormonal and morphological findings. Readers should interpret the reported associations with this caveat in mind. Future prospective studies in this population should incorporate metabolic profiling as a standard component of the diagnostic evaluation.

A further limitation of this study is the lack of available data on sex hormone–binding globulin (SHBG) and assay-specific parameters, which precluded the calculation of free testosterone and the free androgen index and limited a more nuanced assessment of biochemical hyperandrogenism. Additionally, the use of a fixed total testosterone threshold of 55 ng/dL represents a pragmatic approximation, as no universally validated, assay-specific, age- and pubertal-stage-calibrated percentile-based cutoffs for total testosterone in adolescent females currently exist. Sensitivity analyses across thresholds of 40, 50, 55, and 60 ng/dL (**Supplementary Table S1**) confirm that although the absolute prevalence of biochemical hyperandrogenism varies by approximately 12 percentage points across this range, the qualitative conclusion that clinical hyperandrogenism predominates over biochemical hyperandrogenism in adolescent PCOS is robust across all thresholds tested.

## 5 Conclusions

The increasing number of confirmed PCOS cases highlights a growing concern in the pediatric population. Given the limited research on adolescent PCOS, continued engagement of the scientific community is necessary. A better understanding of this population is crucial to refine diagnostic approaches and optimize management strategies. Therefore, it is essential to raise awareness among frontline specialists such as pediatricians and gynecologists, who are often the first to encounter adolescent girls presenting with concerning symptoms, so that appropriate referral and diagnosis can be initiated. A thorough clinical interview during the visit of an adolescent girl reporting potential PCOS-related symptoms should not be underestimated. Given the limited research on this condition in younger adolescents, engagement with the scientific community is essential. Better understanding and characterizing this specific patient group is crucial for further refinement of both diagnostic algorithms and therapeutic strategies.

## Data Availability

The raw data supporting the conclusions of this article will be made available by the authors, without undue reservation.
