# Supplementary material for: Prevalence of polycystic ovary syndrome among adolescents depending on the adopted diagnostic criteria
Source: Front Endocrinol (Lausanne). 2026 Apr 16;17:1785417. doi: 10.3389/fendo.2026.1785417 (PMC13128646; doi:10.3389/fendo.2026.1785417)
Supplement: Supplementary file 1 [file DataSheet1.docx]

**Supplementary Figure 1.** STROBE flow diagram illustrating participant selection and final group allocation. PCOS diagnosis was established according to three different criteria (Ibáñez, Peña, and Rotterdam); therefore, group sizes overlap.
