# Supplementary material for: Prevalence of polycystic ovary syndrome among adolescents depending on the adopted diagnostic criteria
Source: Front Endocrinol (Lausanne). 2026 Apr 16;17:1785417. doi: 10.3389/fendo.2026.1785417 (PMC13128646; doi:10.3389/fendo.2026.1785417)
Supplement: Supplementary file 2 [file Table1.docx]

**Supplementary Table S1**. Testosterone Threshold Sensitivity Analysis - Estimated prevalence of biochemical hyperandrogenism (Part A) and impact on PCOS prevalence (Part B) across four total testosterone thresholds. Estimates for Part A derived from reported group means and SDs (Table 5 of manuscript) using normal approximation; calibrated against manuscript-reported values at T>55 ng/dL (marked ✓). Primary threshold marked ★.

| **Group** | **T > 40 ng/dL** | **T > 50 ng/dL** | **T > 55 ng/dL ★ (primary)** | **T > 60 ng/dL** |
| --- | --- | --- | --- | --- |
| **A. Estimated prevalence of biochemical hyperandrogenism  (% of patients with T above threshold)** | | | | |
| **Ibáñez group (n=132)** | ~78% | ~65% | **54.9% ✓** | ~46% |
| **Peña group (n=151)** | ~78% | ~65% | **55.3% ✓** | ~46% |
| Rotterdam group (n=167) | ~79% | ~66% | **~58%** | ~50% |
| NO PCOS group (n=115) | ~57% | ~40% | **~32% ✓** | ~24% |
| **B. Impact on PCOS prevalence (change in pp vs. primary threshold  T>55 ng/dL)** | | | | |
| *Δ PCOS prevalence Ibáñez criteria* | +1.5–2.0 pp | +0.5–1.0 pp | **Reference** | −1.0–1.5 pp |
| *Δ PCOS prevalence Peña criteria* | +1.5–2.0 pp | +0.5–1.0 pp | **Reference** | −1.0–1.5 pp |

★ = primary threshold used in the manuscript. ✓ = value directly reported in manuscript text. ~ = estimated from reported mean±SD via normal approximation. pp = percentage points. The qualitative finding that biochemical hyperandrogenism is less prevalent than clinical hyperandrogenism is robust across all four thresholds.
