# Supplementary material for: Prevalence of polycystic ovary syndrome among adolescents depending on the adopted diagnostic criteria
Source: Front Endocrinol (Lausanne). 2026 Apr 16;17:1785417. doi: 10.3389/fendo.2026.1785417 (PMC13128646; doi:10.3389/fendo.2026.1785417)
Supplement: Supplementary file 3 [file Table2.docx]

## **Supplementary Table S2**. Sensitivity Analysis: Exclusion by Gynecological Age - PCOS prevalence (Part A) and DHEA-S distribution (Part B) after exclusion of patients with gynecological age < 1 year (n=3) and < 2 years (n=18). All values computed directly from the patient dataset. Reference row (highlighted) = full cohort with available gynecological age data (n=265; 24 patients had missing menarche data).

**Part A PCOS prevalence**

| **Analysis group** | **n** | **Ibáñez PCOS  n (%)** | **Peña PCOS n (%)** | **Rotterdam PCOS  n (%)** |
| --- | --- | --- | --- | --- |
| **Full cohort gynecological age available (primary)** | **265** | **122 (46.0%)** | **140 (52.8%)** | **157 (59.2%)** |
| Excluding gynecological age  < 1 year (n=3 excluded) | 262 | 122 (46.6%) | 140 (53.4%) | 157 (59.9%) |
| Excluding gynecological age  < 2 years (n=18 excluded) | 247 | 119 (48.2%) | 131 (53.0%) | 147 (59.5%) |
| *Δ vs. primary excl. < 1 year* | −3 | +0.6 pp | +0.6 pp | +0.7 pp |
| *Δ vs. primary excl. < 2 years* | −18 | +2.2 pp | +0.2 pp | +0.3 pp |

*pp = percentage points. Positive Δ values reflect the fact that excluded patients did not meet PCOS criteria under any definition, so their removal slightly increases the proportion of PCOS-positive patients.*

**Part B DHEA-S distribution (representative hormonal variable)**

| **Analysis group** | **n** | **DHEA-S median [µg/dL] (IQR)** | **Δ median vs. full cohort** |
| --- | --- | --- | --- |
| **Full cohort gynecological age available** | **255** | **305 (210–418)** | **Reference** |
| Excluding gynecological age  < 1 year | 253 | 305 (209–418) | < 1 µg/dL change |
| Excluding gynecological age  < 2 years | 241 | 307 (212–422) | + 2 µg/dL change |

*DHEA-S selected as representative variable (largest n=255 with valid values). IQR = interquartile range. Exclusion of early post-menarche patients produced negligible changes to the median and IQR.*
