# Supplementary material for: Prevalence of polycystic ovary syndrome among adolescents depending on the adopted diagnostic criteria
Source: Front Endocrinol (Lausanne). 2026 Apr 16;17:1785417. doi: 10.3389/fendo.2026.1785417 (PMC13128646; doi:10.3389/fendo.2026.1785417)
Supplement: Supplementary file 4 [file Table3.docx]

## **Supplementary Table S3.** Sensitivity Analysis: Exclusion of Subjectively Assessed Hirsutism Cases (N=47) - PCOS prevalence and clinical hyperandrogenism rates comparing the full cohort (N=289) with a restricted cohort excluding the 47 patients classified as 'hirsutism present on subjective examination, not assessable due to prior hair removal' (N=242). All values computed directly from the patient dataset.

| **Analysis** | **n** | **Ibáñez PCOS  n (%)** | **Peña PCOS n (%)** | **Rotterdam PCOS  n (%)** |
| --- | --- | --- | --- | --- |
| **Full cohort primary analysis** | **289** | **134 (46.4%)** | **153 (52.9%)** | **170 (58.8%)** |
| Excluding subjective hirsutism (n=47) | 242 | 107 (44.2%) | 122 (50.4%) | 134 (55.4%) |
| *Δ in PCOS prevalence* | — | −2.2 pp | −2.5 pp | −3.4 pp |
| Clinical hyperandrogenism full cohort | 289 | 88.1% | 91.5% | 91.5% |
| Clinical hyperandrogenism excl. subj. hirsutism | 242 | ~78.5% | ~82.0% | ~82.0% |

*pp = percentage points. Clinical hyperandrogenism in restricted cohort = objective mFG ≥ 4 (Peña/Rotterdam) or ≥ 8 (Ibáñez), or severe acne only, without subjective hirsutism classification. ~ = approximate value.*
